# Level of Vegetable Commercialization and Its Determinants Among Smallholder Farmers in Northwestern Ethiopia

**DOI:** 10.1002/fsn3.71545

**Published:** 2026-04-26

**Authors:** Mezgebu Aynalem, Yuning Zhou, Xinyi Liu, Xinru Liu, Guixia Wang, Ao Wang, Gizachew Wosene, Hao Chu

**Affiliations:** ^1^ College of Economics and Management Jilin Agricultural University Changchun China; ^2^ School of Economics and Management Changchun Sci‐Tech University Changchun China

**Keywords:** beta regression, household commercialization index, market oriented production, vegetable commercialization

## Abstract

This study examined the levels of commercialization and determinants for potato, onion, and tomato among smallholder farmers in Northwestern Ethiopia. A total of 310 farm households were selected using a multi stage sampling procedure to collect primary data. The household commercialization index was used to measure the commercialization levels of potato, onion, and tomato. Beta regression and two‐limit Tobit models were employed to investigate the determinants of commercialization levels for these vegetables. The study found that onion and tomato production are highly commercialized, while potato production is more balanced between household consumption and market sales. Factors influencing commercialization levels varied among the vegetables. For onion, factors such as sex of household head, education level, access to market information, and access to credit had positive effect, while distance to extension office and distance to market negatively affected commercialization levels. Potato commercialization was influenced by education level, frequency of extension contacts, and access to credit, with distance to market and cooperatives negatively affecting commercialization. Tomato commercialization was positively affected by farming experience, land size and livestock ownership, market information, number of extension contacts, and access to credit, while household size and distance to market had negative effect. This finding suggests that male headship and better access to agricultural resources lead to an increase in commercialization levels. Moreover, strengthening cooperatives, access to credit and extension services to promote vegetable commercialization in the study area are required.

## Graphical abstract

This study shows that vegetable commercialization varies by crop, with onions and tomatoes highly commercialized while potatoes remain moderately commercialized. Commercialization intensity is strongly influenced by household resources and market access, education, market information, extension contact; land size, livestock ownership, and credit access increase commercialization, whereas larger household size and longer distances to markets, cooperatives, and extension offices reduce it. Strengthening credit services, market information systems, cooperatives, and extension support is vital to enhance commercialization in Northwestern Ethiopia.

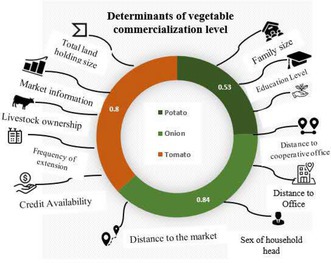

## 1 Introduction

Agriculture remains the backbone of Ethiopia's economy, playing a pivotal role in supporting livelihoods, ensuring food security, and driving economic development. The sector employs approximately 66% of the labor force, contributes about 33% to the country's Gross Domestic Product, and generates over 70% of export earnings (World Bank 2023). Beyond the economic contribution, agriculture is integral to Ethiopia's social fabric, supporting rural community structures, cultural identity, nutrition security, and resilience to climatic and economic shocks (FAO 2021; Neglo et al. 2021). Given that over 80% of people live in rural areas and depend on farming as their primary source of income and subsistence, the performance of the agricultural sector directly shapes national welfare and rural transformation (CSA 2021).

Despite its importance, Ethiopian agriculture is still mainly subsistence oriented. Farmers often produce primarily for household consumption, with limited surplus entering markets. The subsistence orientation limits opportunities for income diversification, technology adoption, and productivity growth. It is widely recognized that transforming agriculture toward a more commercialized and market oriented system is an important pathway for achieving household welfare and sustainable economic growth (Mitiku 2014; Hailua et al. 2015). Agricultural commercialization enhances household welfare by allowing farmers to use resources more efficiently, specialize in high value crops, and integrate into lucrative value chains (Mitiku 2014). Crop production is a source of food and income for smallholder farmers in Ethiopia (Kebede 2020). Hence, transforming subsistence crop production is crucial for achieving food security at the national, regional, and household levels (Merga and Haji 2019).

Vegetable production in Ethiopia is dominated by smallholder farmers and remains an important component in the agricultural landscape of the country (Megerssa et al. 2020). However, despite the agroecological potential of the country, vegetable cultivation remains limited (Milkias and Degefu 2024). National data for 2020/21 show a total of 9,067,870.78 quintals and a yield of 15.88 Qt/ha of vegetables obtained from 243,568.75 ha of cultivated area. In particular, about 419,482.70 quintals and a yield of 65.20 Qt/ha of tomatoes were obtained from 6433.73 ha of land. In addition, onions are grown on 38,952.58 ha with a production of 3,460,480.88 quintals and a yield of 88.84 Qt/ha. Moreover, potatoes cover 85,988.43 ha with a production of 11,418,717.25 quintals and a yield of 132.79 Qt/ha. The Amhara region plays a significant role in national vegetable production, contributing 1,806,557.19 quintals of vegetables from 79,088 ha during the same period (CSA 2021).

Recent evidence highlights that increased commercialization is associated with improved rural household welfare, including higher incomes, asset accumulation, and poverty reduction among smallholder farmers in Ethiopia. For example, smallholder commercialization is found to significantly contribute to rural household income and welfare outcomes in maize producing areas, suggesting commercialization playing a pro poor role in reducing poverty and enhancing livelihood conditions (Geffersa and Tabe‐Ojong 2024). Additionally, studies on teff and other staple crops show the transition toward market oriented production remaining limited but are essential for raising income and food security among smallholders in Ethiopia's northwestern regions (Assefa et al. 2025). Research on cluster farming also demonstrates that organizational innovations can foster teff commercialization and better integrate smallholder farmers into value chains, enhancing livelihoods (Endalew et al. 2024). These recent findings underscore the importance of commercializing agriculture to facilitate structural transformation and sustained rural development in Ethiopia.

Vegetables are grown by smallholder farmers across various agro ecological zones as a source of food, nutrition, and income (Emana et al. 2015; Megerssa et al. 2020; Abe and Tefera 2021). They form an essential livelihood base in many rural communities, especially where market access is favorable. In response to the increasing demand for vegetables in both domestic and international markets, the demand has been on an upward trend (Schreinemachers et al. 2018; Hu et al. 2021). Thus, the government of Ethiopia promotes market oriented vegetable production to capitalize on the roles vegetables play in augmenting household and national economies (Gebru et al. 2019). Particularly in Northwestern Ethiopia, vegetable production is a crucial livelihood activity for smallholder farmers that significantly contribute to food security and income generation. Despite the region's favorable conditions for vegetable production, it is still dominated by a subsistence production system. This may be due to the full engagement of smallholder farmers in a market oriented production system being constrained by several factors. However, these factors have not been well investigated using farm household level data in the region. According to Megerssa et al. (2020) and Joshi et al. (2006), the commercialization of vegetable production is hampered by inefficient farming practices, weak market linkages, and a lack of competitive markets.

Although several studies have examined aspects of horticulture in Ethiopia, important knowledge gaps remain concerning the commercialization of vegetables. For instance, Kebede et al. (2017) studied factors affecting the productivity of potato growers in Ethiopia, but did not assess the household level of commercialization. Tarekegn et al. (2025) studied the implications of post harvest losses on food and nutrition security in smallholder vegetable farming, while Gosa et al. (2024a) examined the effects of vegetable commercialization on household food security. However, they did not address issues of the level of vegetable commercialization and its determinants. Even though the study by Megerssa et al. (2020) examined factors affecting market participation among smallholder vegetable farmers in southwest Ethiopia, it did not address the extent of household level commercialization. There has been little research on vegetable commercialization despite their indispensable role in household and national economies. Available studies on vegetable commercialization are geographically concentrated in central and southern regions and mainly emphasize market participation rather than the level and intensity of commercialization (Tadesse et al. 2018; Gosa et al. 2024b). Moreover most commercialization studies in northwestern Ethiopia focus on staple or cereal crops. For example, (Mihretie 2021) examined wheat commercialization shows low market engagement and unique determinants such as farm size and extension services, but does not address vegetable crops specifically.

Studies regarding the degree of household vegetable commercialization and factors influencing it are limited, especially in the Amhara region. Most of the studies are geographically concentrated and often focus on one type of vegetable crop or general agricultural commodity. Few recent studies have assessed the commercialization of multiple vegetables within one production system, and only a few pieces of research have addressed specific market conditions facing smallholder producers of onion, potato, and tomato in Mecha and Fogera districts. Moreover, the previous studies on smallholder commercialization in Ethiopia have predominantly focused on cereals and pulse crops, leaving the vegetable subsector largely neglected and under researched. Therefore, there is still a gap in current, crop specific and regionally focused evidence concerning the intensity and determinants of vegetable commercialization in northwestern Ethiopia.

Also socioeconomic factors differ from area to area; there is a need for scientific investigations of vegetable commercialization in Ethiopia and the study area to unlock its potential for household and national economies. Therefore, this study seeks to fill the existing research gap and offer meaningful insights for policymakers and other stakeholders in improving the vegetable production system and the commercialization of major vegetables grown by smallholder farmers in the region.

## 2 Theoretical Analysis and Conceptual Framework

### 2.1 Theoretical Analysis

Understanding the commercialization behavior of smallholder vegetable farmers requires an integration of various theoretical perspectives, which bring into focus different dimensions of household decision making and market participation, as well as the wider institutional environment. Agricultural household models, Transaction cost economics; the theory of induced innovation, Resource based theory, and Social Capital Theory collectively constitute a body of knowledge that provides a broader understanding of factors impacting commercialization within a rural environment such as that of Northwestern Ethiopia.

The agricultural household models assume that farm households are simultaneous producers and consumers, thus implying that the degree of participation within the markets and the level of commercialization is driven by preference and the influence of the market itself (Singh et al. 1986). The strengths of the model include its ability to recognize the fact that the basic actor within the farming sector is a household that participates both as a producer and a consumer. Additionally, the model can develop a framework to explain the level of commercialization in markets when prices are attractive. Weaknesses of the model include its failure to account for transaction costs and factors that hinder commercialization in markets.

Transaction Cost Economics further contributes by emphasizing the role of search and transaction costs of negotiation, ongoing monitoring, and the effect of transportation costs when smallholder farmers go into the markets (Williamson 1981). The fact that poor infrastructure and information asymmetry are linked with high transaction costs prevents farmers in Ethiopia's countryside from engaging well within the markets, leading to low levels of commercialization.

The strength of the Transaction Cost Economics theory is that it can explain why farmers fail to commercialize the surplus even when prices are good. However, the weakness of the theory is that it presupposes the existence of functional markets and varying transaction costs, whereas in the Ethiopian context, markets may be nonexistent and characterized by very few participants.

The induced innovation theory by Hayami and Ruttan (1971) describes how changes in resource availability and economic opportunities induce farmers to alter technology and production methods. Faced with either land scarcity or increased market demand, farmers tend to intensify their production and innovate to meet the market demands, whether through better seeds, extension services or more specialized vegetable production. The strength of this theory is that it views commercialization largely as an adjustment to changing market opportunities. Its major weakness is that it assumes access to capital, information, and technology that small scale farmers in Northwestern Ethiopia typically do not have. Nevertheless, the theory helps explain how the production of vegetables shifts from being predominantly subsistence to more commercially oriented and of higher value when market opportunities widen.

Resource based theory provides an important lens through which variability in smallholder commercialization can be understood. According to Barney et al. (2011), households vary depending on the different resources they control, such as land, education, livestock, market information, credit, and extension access, which determine their capacity to produce marketable surplus and engage in commercial agriculture. Commercialization, therefore, is driven by the variability of resources within households, whereas various crops require the synthesis of different resources characterized by the intensity of the resources required based on the level of knowledge and response of the resources used.

Social Capital Theory also contributes to the understanding of the commercialization process, emphasizing the role of networks, trust, norms, cooperatives, and collective action in gaining information, reducing transaction costs, and enhancing bargaining power (Putnam et al. 1994; Narayan and Pritchett 1999). Information regarding prices, demand, and marketing arrangements in rural Ethiopia flows through networks, farmers' groups, and cooperatives. The strength of the theory lies in its recognition that the commercialization of the farming sector is not merely a matter of economic choice but a social phenomenon situated within community structures. However, a weakness of the theory is its acknowledgment that poor households may be left behind due to a lack of social capital. Nonetheless, the theory remains important, explaining the various aspects of the participation of cooperatives, trust in marketing, membership and other factors related to the commercialization of the farming sector.

These various theories, when combined, form a comprehensive foundation for analyzing vegetable commercialization among smallholder farmers. Agricultural household models explain household production consumption dynamics; transaction cost economics highlights market related barriers; induced innovation contextualizes farmers' adaptive responses; resource based theory focuses on the resources they control; while social capital theory identifies how networks reduce information and coordination costs. No single theory can fully explain the process of commercialization, but their integration offers a multidimensional analytical lens suitable for an in depth understanding of the determinants and level of vegetable commercialization in Northwestern Ethiopia.

### 2.2 Conceptual Framework of the Study

Agricultural commercialization refers broadly to the process through which farm households increasingly integrate their production and consumption decisions with the market. Yet, the concept has been defined differently across the literature. Early scholars conceptualized commercialization as the degree to which farm output is destined for sale rather than home consumption (Von Braun and Kennedy 1994). Pingali and Rosegrant (1995) emphasized the transition from subsistence to market orientation through increased participation in input and output markets. Govereh and Jayne (2003) considered commercialization to involve both the specialization of production toward the market and participation in the agricultural value chains. Commercialization has also been considered as not only the sales of goods but also involves changes in the behavior of households, use of inputs, cropping practices, and investment decisions (Gebremedhin and Jaleta 2010). The different notions indicate that commercialization is a complex phenomenon that incorporates production, market factors, and the environment.

A second issue is that the measurement of the degree of commercialization also differs across the various empirical studies. While some studies used a binary variable defined by the extent of participation in the marketing of the produce, such a variable does not consider the degree of participation of small farmers (Strasberg et al. 1999; Martey et al. 2012). Others have used the level of sales but did not consider the level of production of the households (Gebremedhin and Jaleta 2010). Probably the most widely accepted approach is the household commercialization index, which measures the proportion of total production marketed by a household (von Braun et al. 1994). This index captures the level of commercialization and thus allows comparison across households independent of the size of production (Govereh and Jayne 2003). For vegetable crops, given large differences in production and consumption levels, a proportion of marketed surplus over total output has advantages as a measure of commercialization intensity (Gebremedhin and Jaleta 2010).

This study conceptualizes the degree of vegetable commercialization as a fractional measure between 0 and 1, reflecting the share of output sold. This is consistent with empirical evidence showing that commercialization varies not only in participation but also in intensity. This justifies the use of fractional and beta regression models suitable for bounded dependent variables.

**FIGURE 1:**
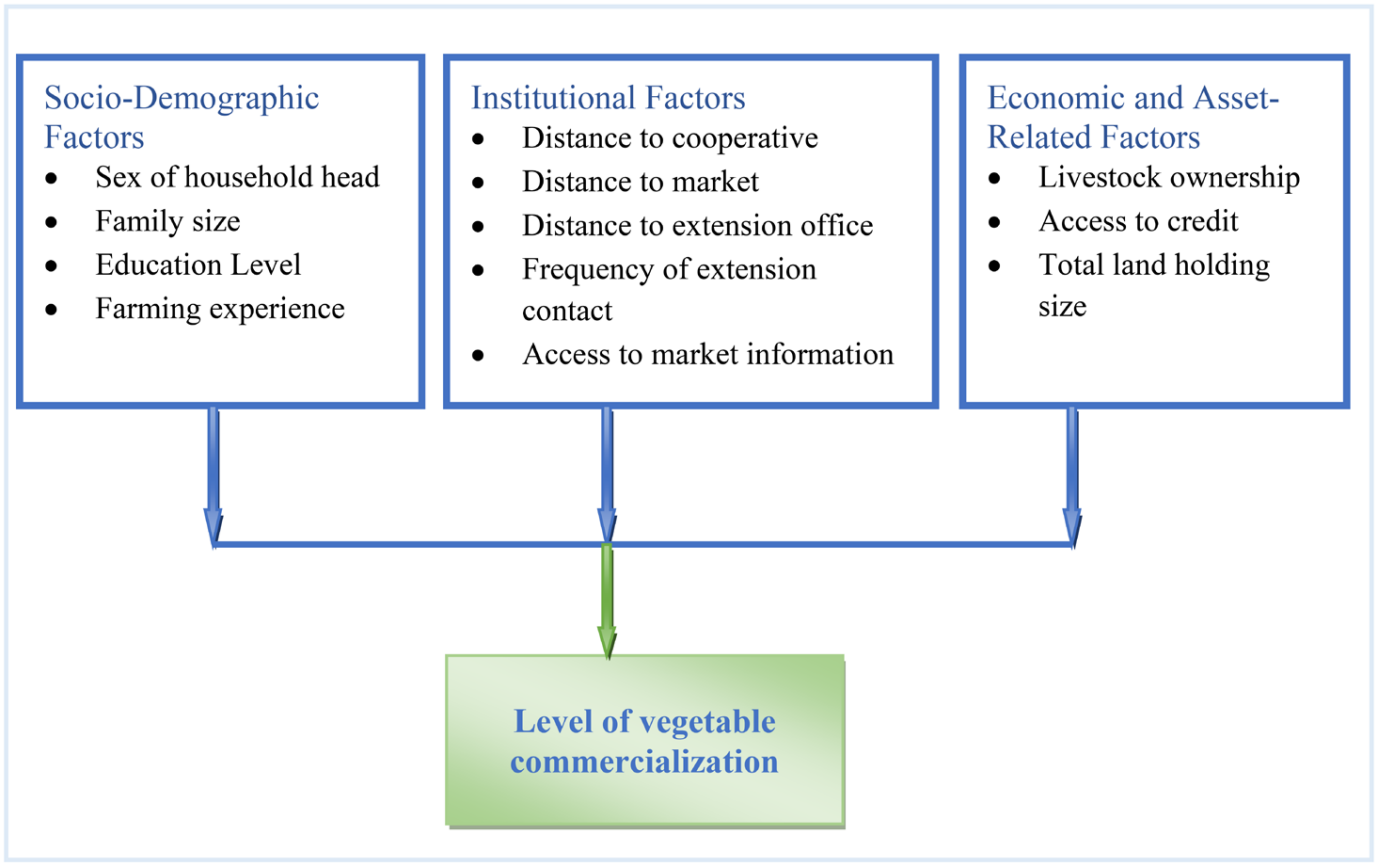
Conceptual framework. Source: Own construction.

Therefore, based on the theoretical perspectives and empirical definitions, the conceptual framework factors influencing the commercialization level of smallholder vegetable farmers are grouped as: socio demographic factors such as sex of the household head, family size, education level, and farming experience; institutional factors such as distance to cooperative office, market, and extension office; frequency of extension contact, and market information; and economic and asset related factors (livestock ownership, credit availability, total land holding size) in Figure 1.

## 3 Materials and Methods

### 3.1 Description of the Study Area

**FIGURE 2:**
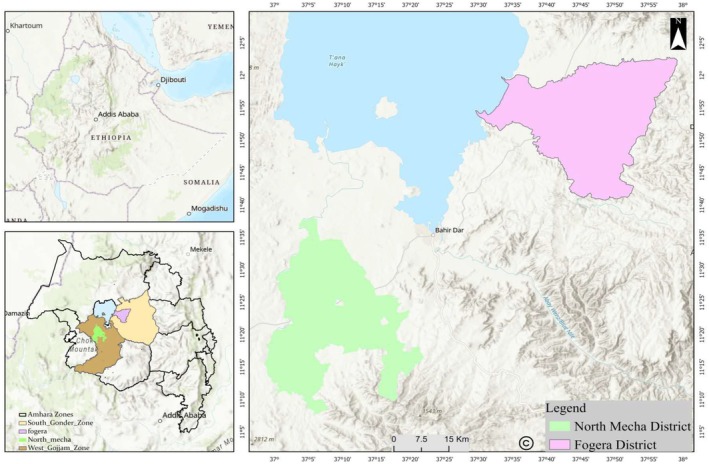
Map of study area. Source: Own construction.

The research was carried out in the Amhara region, Northwestern Ethiopia. In the region, West Gojjam and South Gondar Zones are the centers of vegetable production. Thus, this study targeted Mecha District from West Gojjam Zone and Fogera District from South Gondar Zone (Figure 2).

#### 3.1.1 Fogera District

As noted by Desta et al. (2021), Fogera District is situated at an elevation ranging from 1774 to 2410 m above sea level, lying between latitudes 11°46′ to 11°59′ North and longitudes 37°33′ to 37°52′ East. The district's average annual rainfall is 1216 mm. It is considered one of the surplus producing areas, cultivating a wide variety of annual and perennial crops, including cereals, pulses, oilseeds, vegetables (such as onion, potato, pepper, and tomatoes), spices, and fruits (Fogera District Administration Office 2024).

#### 3.1.2 Mecha District

Mecha District is located at an altitude of 1800 to 2850 m above mean sea level. The district's average annual rainfall is 1200 to 1800 mm. It is also one of the surplus producing districts, with a diverse range of annual and perennial crops such as cereals, pulses, oil crops, vegetables (onion, potato, pepper, and tomatoes), spices, and fruits. Access to irrigation from rivers, such as Gilgel Abay, has also enabled the district to stand out in irrigated vegetable crops studies (Amhara Regional State Meteorological Agency 2023). This district was selected due to its vegetable production potential besides the involvement of smallholder farmers engaged in onion, potato, and tomato production.

### 3.2 Sampling Procedure and Sample Size

A multi stage sampling technique was employed to draw representative samples for this study. The first stage involved the selection of two zones namely West Gojjam and South Gonder Zone, whose relevance to the production of vegetables and importance as suppliers to both local and national markets are considered prominent. The second stage involved the choice of the Mecha district and Fogera district because of the potential for vegetable production and their status as leading vegetable production districts within the Amhara Region as indicated by data from the two zones. Moreover, the two districts contain a large number of smallholder farmers who are mostly engaged in vegetable production making them ideal for assessing commercialization status. Lastly, a total of 310 vegetable producing households were selected using a random sampling technique that is proportionate to the size of the population of each district and the number of vegetable producers. However, since vegetable production in the area was crop specific, not all respondents were growing all three types of vegetables. Consequently, sample sizes specific to crops were: a total of 194 onion growers, 218 potato growers, and 308 tomato growers. The sampling technique used was probability proportional to size.

### 3.3 Methods of Data Collection

The quantitative primary data were collected through face to face interviews using a semi structured questionnaire customized for the study. Before data collection, a pre test was conducted with a small randomly sampled households involved in vegetable production to refine the questionnaire. In addition to household interviews, the study included six Focus Group Discussions, with three held at each study site, involving 8–12 respondents from the community who were vegetable farmers with varying levels of market involvement. The discussions were guided by a checklist. The data collected through these methods were supplemented by twelve Key Informant Interviews with individuals who have extensive knowledge about vegetable production and marketing, including district agriculture office staff, cooperative leaders, and development agents.

### 3.4 Method of Data Analysis

Various analytical techniques were utilized to analyze the collected data, including descriptive statistics, and econometric models.

#### 3.4.1 Descriptive Analysis

Descriptive statistics such as percentages, frequencies, minimum, maximum, mean, and standard deviation were used to summarize and present the characteristics of the sample respondents.

#### 3.4.2 Output Commercialization Index

##### 3.4.2.1 Vegetable Commercialization

This refers to the adoption of a farming system geared toward marketing vegetables. It shows the degree of involvement of smallholder farmers as sellers of the marketed output (Pingali 1997). The level of commercialization of vegetables for each household was quantitatively measured to show the extent to which the vegetables are produced for the market (Jaleta et al. 2009). The level of vegetable commercialization for each of the households was therefore quantitatively measured to indicate the extent to which the production of the vegetables is aimed at the market, similar to the procedure followed in similar studies like (Kabiti et al. 2016). The output commercialization index used in the study calculates the portion of vegetables sold at the market. The formula used, similar to the formula followed by Von Braun and Kennedy (1994), Kabiti et al. (2016) and Sekyi et al. (2020) is as follows:

(1) $$\text{Level of Output commercialization index}=\frac{\text{Quantity sold}\;}{\text{Total quantity produced}}$$

This index varies between 0 and 1, where 0 represents a subsistence farming household (selling nothing), and a household that sells all its production is represented by 1. The level of commercialization is classified into three groups: 0%–25%, 25%–50%, and over 50%, representing subsistence, semi commercial and commercial farmers, respectively (Mamo et al. 2017; Ayele et al. 2021). However, more recent work proposes a four level classification to better reflect the diversity of market engagement: subsistence (0–0.25), low commercialization (0.25–0.50), medium commercialization (0.50–0.75), and high commercialization (> 0.75) (Kissoly et al. 2020; Gosa et al. 2024b). This expanded framework is particularly useful for vegetable producers, whose output varies widely across seasons and households, and it allows a more nuanced assessment of market orientation relevant for the subsequent econometric analysis.

#### 3.4.3 Econometric Model

Fractional response models are appropriate when the dependent variable is expressed as a rate, index, or proportion bounded between 0 and 1 (Baum 2008; Ferrari 2013). Beta regression and fractional response probit models are the most widely used fractional response models. The choice of these models depends on the inclusion and exclusion of the lower and upper bounds of the dependent variable. For example, the beta regression model is appropriate when the dependent variable lies between zero and one instead of including zero and one. In this study, the commercialization index is a fractional variable measured strictly between 0 and 1, with no observations taking the exact values of 0 or 1. Because the dependent variable does not include the boundary values, the beta regression model is theoretically the most suitable model, as it assumes a continuous distribution on the open interval (0, 1) and provides efficient and unbiased estimates for such outcomes. Although the beta regression model has been applied across various fields to tackle similar issues (Pullenayegum et al. 2010; Hunger et al. 2011, 2012; Ünlü and Aktaş 2017), its use in the context of vegetable commercialization remains limited. In this model, the dependent variable follows a beta distribution, characterized by the density function outlined below (Ferrari and Cribari‐Neto 2004; Hunger et al. 2011; Ferrari 2013). The model is specified as follows:

(2) $$f\left(y,\mu ,\phi \right)=\frac{\Gamma \phi }{\left(\Gamma \left(\mu \phi \right)\Gamma \left(1-\mu \right)\phi \right)}y^{\mu \phi -1}{\left(1-y\right)}^{\left(1-\mu \right)\phi -1},0<y<1$$

Here μ represents the expected value of *Y* that is, *E*(*Y*) = μ

The parameter Φ is referred to as the precision parameter, as an increase in its value corresponds to a decrease in the variance of the dependent variable. In particular,

(3) $$\mathrm{Var}\left(Y\right)=\frac{V\left(\mu \right)}{1+\phi }\;\text{where}\;v\left(\mu \right)=\mu \left(1-\mu \right)$$

In the standard beta regression model, only the mean parameter μ of the beta distribution is modeled as a function of the covariates, while the precision parameter Φ is considered a nuisance parameter and $E\left\langle y_{i}|X_{i}\right\rangle =\mu_{i}$ is typically held constant (Hunger et al. 2011).

(4) $$\mu_{i}=\frac{1}{1+\exp\left(-ŋ\right)}=\frac{1}{1+\exp\left(-x^{\prime }\beta \right)}=g\left(x_{i}^{\prime }\beta \right)\forall i$$

where *g(.)* is a known function with 0 < *g(.)* < 1, the model is reformulated using the logit link function as shown below (Baum 2008; Hunger et al. 2012).

(5) $$g\left(\mu_{i}\right)=\ln\left(\frac{\mu_{i}}{1-\mu_{i}}\right)=X_{i}^{\prime }\beta \Rightarrow \ln\left(\frac{\mu_{i}}{1-\mu_{i}}\right)=\beta_{0}+\sum_{i=1}^{n}\beta_{1}X_{1}$$

where *X*_*i*_ represents the vector of explanatory variables, $\beta_{0}$ is the intercept term and $\beta_{i}$ denotes the vector of regression coefficients.

However, in smallholder commercialization contexts, the economic process generating the outcome is bounded by nature. Households may theoretically achieve full subsistence (commercialization index = 0) or complete market orientation (index = 1), even if such cases are not realized in the sample. Because these natural bounds are economically relevant and behaviorally possible for smallholder farmers, it's important to check whether a model that explicitly accounts for censoring at these limits would produce different conclusions. A two‐limit Tobit model was thus estimated alongside the beta regression model. The Tobit model allows for the possibility of outcomes clustering at the lower or upper limits, conceptually plausible in commercialization studies especially in settings where farmers may sell none of their product or, vice versa, almost wholly rely on market sales.

Using both models, therefore, achieves a dual purpose: First, it keeps the econometric strategy consistent with the economic reality of smallholder commercialization, where there are true bounds even if not observed; on the other hand, as robustness check, it verifies whether such results are sensitive to the distribution and treatment of bounds assumed. The two models yielded consistent signs and magnitudes, as well as significance levels of key predictors, thereby corroborating that identified relationships are indeed not model dependent. Beta regression was finally used since it had a better model fit, based on the Akaike Information Criterion (AIC), Bayesian Information Criterion (BIC), and log‐likelihood values, but also for theoretical considerations, given that the outcome variable is fractional and constrained within (0, 1).

## 4 Result and Discussions

### 4.1 Vegetables Commercialization Level

The results revealed that the level of commercialization varies based on the types of vegetable crops. For example in Table 1, the commercialization of potatoes tends to be lower, with the majority of households (134 or 61.47%) at a low commercialization level, with an average commercialization score of 0.45. On the other hand, around 79 households (36.23%) reached a medium level of commercialization, with a mean commercialization score of 0.65. Surprisingly, only 5 farm households (2.3%) were highly commercialized, selling more than 75% of their potato production, with an average commercialization score of 0.79. The overall level of commercialization of potatoes was 0.53, whereby the majority of households were engaged in moderate levels of commercialization. This may be attributed to the fact that potatoes are staple foods and are mainly consumed by farm households rather than sold.

**TABLE 1:** Commercialization level of potato, onion and tomato in the study area.

Vegetable type	Commercialization level	Frequency	Mean	Std. Dev.	Minimum	Maximum
Potato	Low (25%–50%)	134 (61.47%)	0.45	0.08	0.29	0.50
	Medium (50%–75%)	79 (36.23%)	0.65	0.05	0.56	0.75
	High (> 75%)	5 (2.3%)	0.79	0.04	0.76	0.86
	Total	218	0.53	0.13	0.29	0.86
Onion	Low (25%–50%)	5 (2.58%)	0.47	0.03	0.42	0.5
	Medium (50%–75%)	30 (15.46%)	0.66	0.08	0.53	0.75
	High (> 75%)	159 (81.96%)	0.88	0.05	0.75	0.97
	Total	194	0.84	0.12	0.42	0.97
Tomato	Low (25%–50%)	2 (0.65%)	0.44	0	0.44	0.44
	Medium (50%–75%)	81 (26.3%)	0.68	0.05	0.53	0.75
	High (> 75%)	225 (73.05%)	0.85	0.05	0.77	0.98
	Total	308	0.80	0.09	0.44	0.98

In the case of onions, commercialization trends show a sharp contrast, as a majority of households (81.96%) are registering high levels of commercialization (> 75%) with an average commercialization index of 0.88. This shows that onions are produced mainly for the market and consumed through a small proportion. Very few (2.58%) are registering low levels of commercialization with an average index of 0.47, while those classified as medium commercialized (15.46%) have an average index of 0.66. The overall commercialization index for onions is 0.84, which reflects a strong orientation toward the commercial sector. This may be due to low requirement as a spice at household level and mainly produced for the market. During the high production season, onion prices are extremely low.

For tomatoes, commercialization is mainly at a high level, with 73.05% recording an average index of 0.85. This high market orientation is mainly due to the perishable nature of tomatoes, which requires quick sales. A moderate level of commercialization is represented by 26.3% of the households, with an average index of 0.68, while only 0.65% recorded a low level of commercialization, with a fixed index of 0.44. The overall average index of commercialization for tomatoes is 0.80, indicating a high level of commercialization.

Overall, for potato growing households, most were falling into the low to medium levels of commercialization, and a few at high levels. Onions and tomatoes show high levels of commercialization, possibly high demand, and high perishability while potatoes are moderately commercialized, as they are mainly used for home consumption.

### 4.2 Vegetables Production

The data on vegetable production, consumption, and sales for onions, potatoes, and tomatoes reflect significant variations in the utilization patterns of these vegetables by different households, depending on demand and other factors in Table 2.

**TABLE 2:** Amount of vegetable production, consumed, and sold in quintal.

Vegetable type	Production in quintal		Mean	Dev. Std.	Min	Max
Onion	Amount of produced	194	29.8	27.27	3.75	162
	Amount of consumed	194	3.51	2.68	0.5	15
	Amount of sold	194	26.28	25.24	1.75	152
Potato	Amount of produced	218	18.2	8.83	6	60
	Amount of consumed	218	7.77	2.29	2	14
	Amount of sold	218	10.45	7.36	2	46
Tomato	Amount of produced	308	13.54	8.13	2.5	48
	Amount of consumed	308	2.41	1.59	0.1	14
	Amount of sold	308	11.13	7.07	2	40

The amount of onion produced is high, with an average of 29.8 quintals, and a large standard deviation of 27.27. The amount consumed is small, with an average of 3.51 quintals, compared to the amount sold, which is 26.28 quintals. Relatively low household requirement for consumption (used as a spice) and, a relatively lower shelf life indicate that onions are mainly produced for sale rather than household consumption. In the case of potatoes, the amount produced per household is lower, on average 18.2 quintals, with less variability than onions. The amount sold by households is 10.45 quintals on average, which is higher than the amount consumed (7.77 quintals). The proportion sold is smaller than onions, possibly because potato are used as food while onions are used as food seasoning.

For tomatoes, the average production is 13.54 quintals. The amount of home consumption is relatively low, at 2.41 quintals, indicating that this commodity is more oriented toward the market. The quantity sold (11.13 quintals) is almost equal to the total production, suggesting that tomatoes are mainly produced for the market. The perishable nature of tomatoes may drive households to sell them quickly to avoid losses on this staple commodity. Overall, onions and tomatoes are more market driven and commercially oriented, while a larger proportion of potatoes is retained for home consumption.

### 4.3 Determinants of Potato, Onion and Tomato Commercialization Level

This study used beta regression and two‐limit Tobit models to determine the best model that explains the relationship between the dependent and explanatory variables. The result in Table 3 shows that the AIC, BIC, and log likelihood values indicate that the beta regression model is better than the two‐limit Tobit model for this data set. The results of the beta regression model were further validated using the two‐limit Tobit model to ensure the robustness of the findings. The parameter estimates in both models are consistent in terms of significance, with similar predictors being significant in both models for each crop. The parameter signs and estimates were also largely comparable across models, indicating the reliability of the identified relationships. In conclusion, the beta regression model was deemed more appropriate and in line with the bounded nature of the dependent variables.

**TABLE 3:** Parameter estimates of beta regression and two‐limit Tobit models.

Variables	Parameter estimates of beta regression model			Parameter estimates of two‐limit Tobit model		
	Onion	Potato	Tomato	Onion	Potato	Tomato
Sex of household head	0.024*	−0.001	−0.008	0.031**	−0.002	−0.008
	(0.013)	(0.018)	(0.010)	(0.015)	(0.018)	(0.011)
Farming experience	0.001	−0.001	0.002***	−0.002	−0.000	0.002**
	(0.001)	(0.001)	(0.001)	(0.001)	(0.001)	(0.001)
Household size	0.001	−0.004	−0.016***	0.001	−0.004	−0.013**
	(0.006)	(0.008)	(0.005)	(0.007)	(0.008)	(0.005)
Education level	0.005*	0.002***	−0.001	0.006**	0.002***	−0.001
	(0.003)	(0.001)	(0.002)	(0.003)	(0.001)	(0.002)
Distance to market	0.001	−0.002***	−0.001**	0.000	−0.002***	−0.001*
	(0.001)	(0.001)	(0.001)	(0.001)	(0.000)	(0.000)
Distance to cooperative office	0.000	−0.007**	−0.001	0.000	−0.008**	−0.001*
	(0.001)	(0.004)	(0.001)	(0.001)	(0.004)	(0.000)
Distance to extension office	−0.002***	0.001	0.001	−0.002***	0.000	0.000
	(0.001)	(0.001)	(0.001)	(0.001)	(0.001)	(0.001)
Total land holding size	0.004	0.014	0.022*	0.002	0.012	0.024**
	(0.014)	(0.021)	(0.011)	(0.016)	(0.022)	(0.011)
Livestock ownership	−0.003	−0.005	0.009***	−0.005	−0.005	0.010***
	(0.003)	(0.004)	(0.002)	(0.003)	(0.004)	(0.002)
Market information	0.035**	−0.001	0.048***	0.051**	−0.001	0.057***
	(0.014)	(0.016)	(0.010)	(0.016)	(0.016)	(0.011)
Frequency of extension contact	−0.004	0.014***	0.004***	−0.004	0.015***	0.003***
	(0.004)	(0.005)	(0.001)	(0.005)	(0.005)	(0.001)
Access to credit	0.048***	0.034*	0.023**	0.059***	0.033*	0.018*
	(0.015)	(0.019)	(0.010)	(0.017)	(0.019)	(0.011)
Constant	0.596	−0.002	−0.040	0.644***	0.508***	0.590***
	(0.450)	(0.345)	(0.297)	(0.069)	(0.086)	(0.047)
Log likelihood	211.863	170.238	358.98	173.665	167.906	336.75
AIC	−399.73	−316.48	−693.96	−323.33	−311.81	−649.50
BIC	−360.45	−275.87	−649.16	−284.05	−271.20	−604.70
LR chi2 (12)	48.37	49.06	86.65	58.98	48.52	82.90
Prob > chi2	0.0000	0.0000	0.0000	0.0000	0.0000	0.0000
Sigma				0.099***	0.112***	0.081***
				(0.005)	(0.005)	(0.003)
Observations	194	218	308	194	218	308

*Note:* Standard errors in parentheses ***p < 0.01, **p < 0.05, *p < 0.1.

The model results in Table 3 indicated that households headed by males had a positive effect on the commercialization of onions (*p* < 0.1). This result suggests that males are more likely to engage in a market oriented onion production system may be male headed households have greater access to resources and market networks. According to Quisumbing and Pandolfelli (2008), in the theory of gender differentiated resource allocation, males are usually better endowed with resources such as land, credit, and extension services in agricultural contexts, enabling them to be more productive and market oriented. This effect is consistent with resource based theories, which note that resource ownership and control are crucial for market access. This finding is consistent with previous studies (Alene et al. 2008; Gosa et al. 2024b) which noted that male headed households are more like to engage in commercial agriculture because of their advantage in negotiating with potential value chain actors.

Similarly, farming experience positively and significantly influenced tomato commercialization at *p* < 0.01. As indicated by the human capital theory and further supported by the work of Schultz (1961), farming knowledge and experience accumulated over time improve the farmer's ability to make more efficient decisions on resource management, leading to improved productivity and marketed surplus. With experience, farmers are usually better managers of risk and optimizers of input use, translating into better market integration. Studies by Perressim and Batalha (2024), showed that farming experience is related to more efficient utilization of the market and response to market signals. This finding is also supported by Endalew et al. (2020).

Household size has a negative and significant effect on tomato commercialization (*p* < 0.01). The subsistence oriented household model supports the negative effect of household size on tomato commercialization. This can be explained by the fact that larger households typically have higher internal consumption needs, which reduces the surplus available for sale. In subsistence settings where household food security is of utmost importance, production is often geared toward meeting family consumption before market participation. According to Singh et al. (1986), larger households often give priority to satisfying consumption needs over market sales, especially with perishable crops like tomatoes with high immediate consumption value and high risk of spoilage. Larger families, according to studies such as Asfaw and Shiferaw (2010), retain a greater share of output for household consumption, thus reducing marketable surplus in line with the agricultural household model, where the allocation of labor and output is first directed to household home consumption, limiting commercialization.

On the other hand, education level positively affected onion commercialization (*p* < 0.1) and potato commercialization (*p* < 0.01), which can be viewed from a human capital and adoption theory perspective. From a human capital perspective, education enhances farmers' knowledge, skills, and decision making ability to better manage their farms and respond to market opportunities. According to Rogers (2003), education enhances farmers' ability to obtain and interpret market information for action, which relates to notions in adoption theories. The educated farmer will more likely adopt improved technology and respond to market needs and demands; this increases output quality and quantity, an essential prerequisite for successful commercialization. This agrees with the findings of Endalew et al. (2020) and Gosa et al. (2024b), wherein education improves market participation as farmers are able to better understand the requirements of the market and use modern farming technologies.

Distance to the nearest market negatively affected the commercialization of potatoes (*p* < 0.01) and tomatoes (*p* < 0.05). Market distance has a negative effect on potato and tomato commercialization based on its underpinning in spatial transaction cost theory. The higher the distance between a farm and market, the greater the transportation costs, which reduce the effective price received by farmers, thus discouraging market participation (Key et al. 2000). According to Barrett (2010), distance increases transaction costs and information asymmetry, resulting in weak incentives for market oriented production, especially for perishable goods like tomatoes. This implies that proximity to a market is crucial for commercialization, especially for perishable crops supported by Endalew et al. (2020). Distance to the cooperative office negatively influences potato commercialization (*p* < 0.05). Cooperatives reduce transaction costs by providing inputs, credit, and collective bargaining power, which is particularly beneficial for smallholder farmers (Fischer and Qaim 2012). This aligns with the findings of Bernard et al. (2008) that membership in cooperatives enhances commercialization by improving access to markets and inputs, especially for high input crops like potatoes.

Frequency of extension contact positively affected the commercialization of potatoes (*p* < 0.01) and tomatoes (*p* < 0.01). This suggests that extension services promote commercialization by increasing smallholder farmers' productivity and marketed surplus in agrarian countries like Ethiopia. As expressed earlier, frequency of extension contact influences both potato and tomato commercialization, which can be justified using technology adoption and diffusion theory. According to Anderson and Feder (2004), extension services provide technical support and market information that enable farmers to adopt the best practices in farming, increasing the level of productivity and marketed surplus. Furthermore, Endalew et al. (2020) and Davis et al. (2012) and Gosa et al. (2024b) reported that frequent extension contact significantly enhances market participation by improving farmers' ability to produce high quality and high quantity outputs in crops like tomato and potato, which are characterized by high demands for production.

Access to credit significantly and positively affected the commercialization of onions at *p* < 0.01, potatoes at *p* < 0.1, and tomatoes at *p* < 0.05. Credit access increases the resilience of farmers and their ability to invest; hence it is one of the important aspects of market engagement. According to Feder (1985), access to credit relaxes the financial constraint that hinders investing in inputs and managing production risks, hence increasing the marketable surplus. The same result has also been supported by Ayele et al. (2021) and Gosa et al. (2024b), indicating that access to credit reduces liquidity constraints, hence enabling higher production and market participation.

Although a number of the explanatory variables are common across the three crops, their statistical significance varies across onion, potato, and tomato commercialization. These differences reflect not only the inherent production and market characteristics of each crop but also crop specific sample attributes.

## 5 Conclusion

The study reveals that commercialization levels vary significantly across vegetable types, reflecting differences in households' market engagement and resource allocation strategies. Potatoes, with an aggregate commercialization index of 0.53, are primarily produced for consumption and limited sales. Their moderate commercialization level indicates that while market engagement is present, household home consumption remains a priority. Onions and tomatoes exhibit higher levels of commercialization, with aggregate commercialization index of 0.84 and 0.80, respectively may be driven by relatively high market demand, profitability, and established supply channels. This variation underscores that households' commercialization decisions are influenced by crop characteristics such as perishability, market accessibility, and local demand dynamics.

The study found that various factors such as household size, education level, access to credit, market information, distance to market, and frequency of extension contact playing significant roles in influencing the commercialization of onions, potatoes, and tomatoes in the study area. Understanding and addressing these factors can help to improve the commercialization of these crops among smallholder farmers.

Both the descriptive and econometric model results provide concrete evidence to promote onion, potato and tomato commercialization. Thus, by addressing the factors affecting commercialization, policymakers and other stakeholders can foster onion, potato and tomato commercialization to improve the welfare of smallholder farmers in the study area. For example, access to market information significantly influences commercialization, especially for high demand crops like tomatoes and onions; it is recommended to improve information dissemination systems. Establishing local market information centers and using digital platforms can provide timely price and demand data to farmers. Moreover, credit access positively affected commercialization, suggesting that increased financial access can empower farmers to invest in input intensive crops. Developing more accessible and flexible agricultural credit facilities tailored to smallholder farmers could encourage higher commercialization levels across vegetable types.

Targeted interventions such as better storage facilities, improved access to inputs, and training on market diversification could help increase market engagement. This would allow farmers to capitalize on the vegetable market potential without compromising food security. Proximity to markets is also a significant constraint for commercialization, particularly for perishable crops. Enhancing rural infrastructure, such as transport networks, and establishing local marketplaces could reduce costs and logistical challenges, allowing more farmers to engage in market oriented production. Besides, male headed households are more commercially engaged due to greater access to resources. Gender inclusive policies that enhance women's access to resources like credit, land, and extension services can foster a more equitable environment for commercialization among female headed households.

## Author Contributions

**Mezgebu Aynalem:** conceptualization, investigation, methodology, validation, visualization, software, formal analysis, data curation, supervision, resources, writing – original draft, writing – review and editing. **Yuning Zhou:** project administration, validation, writing – review and editing. **Xinyi Liu:** methodology, validation, writing – review and editing. **Xinru Liu:** data curation, writing – review and editing. **Guixia Wang:** conceptualization, investigation, methodology, visualization, writing – review and editing, formal analysis, supervision. **Ao Wang:** conceptualization, methodology, visualization, supervision, writing – review and editing. **Gizachew Wosene:** writing – review and editing, methodology, validation, software, formal analysis. **Hao Chu:** investigation, validation, writing – review and editing, software, supervision, formal analysis.

## Funding

The authors have nothing to report.

## Ethics Statement

No human or animal tissues were used in this study. Procedurally, the similarity index was measured using iThenticate score before journal selection. Then, the plagiarism result was evaluated and confirmed before submission to the journal. Therefore, the manuscript was seriously evaluated to meet ethical standards before submission.

## Conflicts of Interest

The authors declare no conflicts of interest.

## Data Availability

The data used for this study are available from the corresponding author on reasonable request.
